# Supplementary material for: Patterns of Intron Gain and Loss in Fungi
Source: PLoS Biol. 2004 Nov 30;2(12):e422. doi: 10.1371/journal.pbio.0020422 (PMC532390; doi:10.1371/journal.pbio.0020422)
Supplement: Table S1 — Also available at http://genes.mit.edu/NielsenEtAl/. (4.3 MB ZIP). [file pbio.0020422.st001.zip › NielsenEtAl/html/318.html]

AN9498.1.NCU08664.1.MG01886.1.FG04190.1


```
 CLUSTAL W (1.82) Multiple Sequence Alignments - Introns Inserted


Sequence 1: NCU08664.1	276 aa
Sequence 2: FG04190.1	268 aa
Sequence 3: MG01886.1	283 aa
Sequence 4: AN9498.1	275 aa
Alignment Length: 299 aa
Number Identitical Residues: 88 aa
Alignment Score (without introns) 4716


MG01886.1 	MAADTS---SDSKKGTEPPVDTADDEAIPAR-----FAPEEEK0ELVQESNAAKTE~ANS
NCU08664.1	MSSKTPDDKLEQKKVKDPQAEEENDESKQAD---IKFSPEEEA~----------AK1ANA
FG04190.1 	MSSSEP---INDDVVKKPDAGEKSDAGDPADGEELRFTPEEE-~------------~---
AN9498.1  	MPPDTSSASNGRDLTNHAGSDTENEDEVFHD---ARFPPEEET0RLLAESHSLKAE~ANN
          	*... .. .   .  ...     .:           *.****      .: : .:. :. 

MG01886.1 	LFTAGK~CDAALDKYNQAIAVCPNYLDYEVAVLKSNVAACYLKLKEWKEATTAATESLDR
NCU08664.1	LFTSGK~YDAALNKYDEAIAVCPNYLDYPLAVLRSNVAACHLMLEAWKDAITHATKALDL
FG04190.1 	-----E0YQDALNKYDDAINSCPKYLHYPRAVIYSNIAACHIQLEDWKEAIKSASDSLRS
AN9498.1  	LYSAAS~YSQAISTYDRALASCPSYLDYEIAVVRSNMSACYLKLGDWKAAVDSANACIEG
          	  ::..  . *:..*: *:  **.**.*  **: **::**:: *  ** *   *. .:  

MG01886.1 	LDKVEKAD----AQEAEVEKAAEAEELADVEETIVSAGAAQAGPALPTKAETPAQSARRQ
NCU08664.1	LDKLERED--------KLAAEKEEKEKEDVEEEIVSAGAAKAGPAVPV-TETEAQIARQK
FG04190.1 	LEKLEQNDPRLQHDGKKDGEEKSKKEADDVEEEIISSGASRAAPAPES-VDEEVKKLKVN
AN9498.1  	LDRVVPPS------QPEDGKEGAAKEADSVVEIPVDDEVNEQAALQRIKENDERKR----
          	*:::   .        :       :*  .* *  :.  . . ..    . :   :     

MG01886.1 	RAEDVRRIRAKALMRRARARSEAGGWSNLAGAEEDYKLLSKMSN-LGPADRKVVQAQLRL
NCU08664.1	RLEDIARIRAKALLRRARARSELGGWSTLEGAIEDYKKLSAMTN-LTATDRKLVQAQLRA
FG04190.1 	----IQRIRCKALMRRARGRTEAGGWHNLAGAEEDCKALAAKPESLTPADLRVVQKQLRE
AN9498.1  	---DVARIRAKALMRRARAKSELGGWANLQGAEEDYKLLAGMEN-LPPSDRKVVQKALRE
          	   .: ***.***:****.::* *** .* ** ** * *:   : * .:* ::**  ** 

MG01886.1 	LPAQVKAAQEKETAEMWGKLKE0LGNGILKPFGLSTDNFKMEQDPKTGGYSMNFQQNGSK
NCU08664.1	LPPRAKAAQEKETAEMWGKLKD~LGNGLLKPFGLSTDHFKMVKDEKTGGYSMNFQGGGGE
FG04190.1 	LPPRVKAAQEKEMGEMWGKLKD~LGNGILKPFGLSTNNFQMVKDENTGGYSMNFQPGGKS
AN9498.1  	LPARISKAKEVEMGEMMGKLKE0LGNGILKPFGLSTDNFKFVQDPKTGGYSVNFSS----
          	**.: . *:* * .** ****: ****:********::*:: :* :*****:**.     

MG01886.1 	---
NCU08664.1	GKK
FG04190.1 	Q--
AN9498.1  	---
          	
```
